# Appropriate targeting of artemisinin‐based combination therapy by community health workers using malaria rapid diagnostic tests: findings from randomized trials in two contrasting areas of high and low malaria transmission in south‐western Uganda

**DOI:** 10.1111/tmi.12748

**Published:** 2016-08-09

**Authors:** Richard Ndyomugyenyi, Pascal Magnussen, Sham Lal, Kristian Hansen, Siân E. Clarke

**Affiliations:** ^1^ Vector Control DivisionMinistry of HealthKampalaUganda; ^2^ Centre for Medical ParasitologyDepartment for Immunology and MicrobiologyFaculty of Health and Medical SciencesUniversity of CopenhagenCopenhagenDenmark; ^3^ Department of Disease ControlFaculty of Infectious and Tropical DiseasesLondon School of Hygiene and Tropical MedicineLondonUK; ^4^ Department of Global Health and DevelopmentFaculty of Public Health and PolicyLondon School of Hygiene and Tropical MedicineLondonUK

**Keywords:** Rapid diagnostic test (RDT), artemisinin‐based combination therapy (ACT), community health workers (CHWs), appropriately targeted treatment, Uganda, community case management, test de diagnostic rapide (TDR), combinaison thérapeutique à base d'artémisinine (ACT), agents de santé communautaire (ASC), traitement ciblé approprié, Ouganda, prise en charge communautaire des cas

## Abstract

**Objective:**

To compare the impact of malaria rapid diagnostic tests (mRDTs), used by community health workers (CHWs), on the proportion of children <5 years of age receiving appropriately targeted treatment with artemisinin‐based combination therapy (ACT), vs. presumptive treatment.

**Methods:**

Cluster‐randomized trials were conducted in two contrasting areas of moderate‐to‐high and low malaria transmission in rural Uganda. Each trial examined the effectiveness of mRDTs in the management of malaria and targeting of ACTs by CHWs comparing two diagnostic approaches: (i) presumptive clinical diagnosis of malaria [control arm] and (ii) confirmatory diagnosis with mRDTs followed by ACT treatment for positive patients [intervention arm], with village as the unit of randomisation. Treatment decisions by CHWs were validated by microscopy on a reference blood slide collected at the time of consultation, to compare the proportion of children <5 years receiving appropriately targeted ACT treatment, defined as patients with microscopically‐confirmed presence of parasites in a peripheral blood smear receiving artemether‐lumefantrine or rectal artesunate, and patients with no malaria parasites not given ACT.

**Results:**

In the moderate‐to‐high transmission area, ACT treatment was appropriately targeted in 79.3% (520/656) of children seen by CHWs using mRDTs to diagnose malaria, *vs*. 30.8% (215/699) of children seen by CHWs using presumptive diagnosis (*P* < 0.001). In the low transmission area, 90.1% (363/403) children seen by CHWs using mRDTs received appropriately targeted ACT treatment *vs*. 7.8% (64/817) seen by CHWs using presumptive diagnosis (*P* < 0.001). Low mRDT sensitivity in children with low‐density parasitaemia (<200 parasites/*μ*l) was identified as a potential concern.

**Conclusion:**

When equipped with mRDTs, ACT treatments delivered by CHWs are more accurately targeted to children with malaria parasites. mRDT use could play an important role in reducing overdiagnosis of malaria and improving fever case management within iCCM, in both moderate‐to‐high and low transmission areas. Nonetheless, missed treatments due to the low sensitivity of current mRDTs in patients with low parasite density are a concern. For community‐based treatment in areas of low transmission and/or non‐immune populations, presumptive treatment of all fevers as malaria may be advisable, until more sensitive diagnostic assays, suitable for routine use by CHWs in remote settings, become available.

## Abstract (fr)

**Objectif:**

Comparer l'impact des tests de diagnostic rapide (TDR) du paludisme, utilisés par les agents de santé communautaires (ASC), sur la proportion d'enfants <5 ans qui reçoivent un traitement de combinaison à base d'artémisinine (ACT) ciblé de manière appropriée pour le traitement présomptif.

**Méthodes:**

Des essais randomisés en grappes ont été menés dans deux zones rurales contrastées de transmission modérée à élevée et de transmission faible du paludisme en Ouganda. Chaque essai a examiné l'efficacité des TDR dans la prise en charge du paludisme et le ciblage de l'ACT par les ASC en comparant deux approches diagnostiques: (1) le diagnostic présomptif clinique du paludisme [bras témoin] et (2) le diagnostic de confirmation avec les TDR suivi d'un traitement ACT pour les patients positifs [bras d'intervention]; avec le village comme unité de randomisation. Les décisions de traitement par les ASC ont été validées par la microscopie sur un frottis de sang de référence recueilli au moment de la consultation, pour comparer la proportion d'enfants <5 ans qui reçoivent un traitement ACT ciblé de façon appropriée, défini comme les patients avec une présence de parasites confirmée microscopiquement dans un frottis de sang périphérique, recevant un ACT ou de l'artésunate par voie rectale, et les patients sans parasites du paludisme qui ne reçoivent pas d'ACT.

**Résultats:**

Dans la zone de transmission modérée à élevée, le traitement ACT a été ciblé de manière appropriée chez 79,3% (520/656) des enfants vus par les ASC en utilisant les TDR pour diagnostiquer le paludisme, contre 30,8% (215/699) des enfants vus par les ASC en utilisant du diagnostic présomptif (*P* < 0,001). Dans la zone de transmission faible, 90,1% (363/403) des enfants vus par les ASC utilisant les TDR ont reçu de manière appropriée le traitement ciblé ACT contre 7,8% (64/817) des enfants vus par les ASC en utilisant le diagnostic présomptif (*P* < 0,001). Une sensibilité faible pour les TDR chez les enfants avec une faible densité de parasitémie (<200 parasites/μl) a été identifiée comme un problème potentiel.

**Conclusion:**

Lorsqu’équipés de TDR, les traitements ACT délivrés par les ASC sont ciblés avec plus de précision aux enfants avec des parasites du paludisme. L'utilisation des TDR pourrait jouer un rôle important dans la réduction du surdiagnostic du paludisme et dans l'amélioration de la prise en charge des cas de fièvres au sein de la prise en charge intégrée communautaire des cas, à la fois dans les deux zones de transmission modérée à élevée et faible. Néanmoins, les traitements manqués en raison de la faible sensibilité des TDR actuels chez les patients présentant une densité parasitaire faible restent une préoccupation. Pour le traitement à base communautaire dans les zones de faible transmission et/ou à populations non immunes, le traitement présomptif de toutes les fièvres comme étant le paludisme peut être conseillé, jusqu’à ce que des tests de diagnostic plus sensibles, convenant à une utilisation de routine par les ASC dans des régions éloignées, deviennent disponibles.

## Abstract (es)

**Objetivo:**

Comparar el impacto de las pruebas de diagnóstico rápido de malaria (PDRms) utilizadas por trabajadores sanitarios comunitarios (TSC) sobre la proporción de niños <5 años que recibieron adecuadamente la terapia de combinación basada en la artemisinina (TCA), comparado con tratamiento presuntivo.

**Métodos:**

Se realizaron ensayos aleatorizados en conglomerados en dos áreas rurales contrastables, con intensidades de transmisión de malaria moderadamente‐alta y baja de Uganda. Cada ensayo examinó la efectividad de PDRms en el manejo de la malaria y la elección de TCA por TSC comparando dos metodologías diagnósticas: (1) diagnóstico clínico presuntivo de malaria [brazo control] y (2) diagnóstico confirmatorio con PDRms seguido por tratamiento con TCA para pacientes positivos [brazo de intervención]; con el poblado como unidad de aleatorización. Las decisiones sobre el tratamiento por los TSCs se validaron mediante microscopía de extensiones de sangre de referencia recogidas en el momento de la consulta, para comparar la proporción de niños <5 años recibiendo un TCA adecuadamente elegido, (definidos como pacientes con confirmación microscópica de presencia de parásitos en una extensión de sangre periférica y recibiendo tratamiento con TCA o artesunato rectal), y aquellos sin parásitos de malaria que no recibieron TCA.

**Resultados:**

En el área de transmisión moderadamente‐alta, el tratamiento con TCA estaba apropiadamente elegido para un 79.3% (520/656) de los niños visitados por los TSCs utilizando PDRms para diagnosticar la malaria, vs. 30.8% (215/699) de los niños visitados por TSCs y utilizando diagnóstico presuntivo (*P *< 0.001). En el área de baja transmisión, 90.1% (363/403) de los niños visitados por TSCs utilizando PDRms recibieron un tratamiento con TCA elegido apropiadamente vs. 7.8% (64/817) de los visitados por TSCs utilizando un diagnóstico presuntivo (*P *< 0.001). Se identificó como una preocupación potencial la baja sensibilidad a las PDRms en niños con una parasitemia de baja intensidad.

**Conclusión:**

Con el uso de PDRms, los tratamientos con TCA entregados por TSCs son más precisos en niños con parásitos de malaria. Los PDRms podrían jugar un papel importante para reducir el sobre diagnóstico de malaria y mejorar el manejo de casos de fiebre dentro del iCCM, tanto en áreas de transmisión moderada‐a‐alta como de baja transmisión. Sin embargo, los tratamientos perdidos en pacientes con una baja densidad de parásitos, debido a la baja sensibilidad de los PDRms actualmente disponibles, son preocupantes. Para tratamientos basados en la comunidad en áreas de baja intensidad de transmisión y/o poblaciones no inmunes, el tratamiento presuntivo como malaria de todas las fiebres sería recomendable hasta que haya disponibilidad de pruebas diagnósticas más sensibles, y adecuadas para un uso rutinario por TSCs en emplazamientos remotos.

## Introduction

Early diagnosis and effective case management is a central component of the current malaria control strategy 1, 2, yet millions live at or beyond the periphery of the health system. Improving access to care for childhood infectious diseases by bringing treatment closer to the community is an attractive option, especially in rural settings where distance, cost and infrastructural challenges of primary healthcare centres limit access to expert services 3. Targeting artemisinin‐based combination therapy (ACT) to those who are parasitologically confirmed malaria cases is critical in reducing misdiagnosis and overuse of these expensive drugs and may play an important role in the financial sustainability of community‐based programmes 4, 5, 6. The World Health Organization (WHO) now recommends universal access to malaria diagnostics 7, and at the moment, antigen‐based rapid diagnostic tests (mRDTs) are the only feasible test at community level. The case for their use rather than presumptive treatment is strong, due to lack of accurate algorithm‐based diagnosis 8 and negative consequences of overuse of antimalarials 6. Although there is evidence that mRDTs and ACTs can be successfully deployed at community level 9, 10, there is little information on whether their use improves the targeting of ACTs to malaria patients at that level, especially in low transmission settings. We conducted a cluster‐randomized controlled trial to compare the impact of mRDTs, used by CHWs, on the proportion of children <5 years of age receiving appropriately targeted treatment with ACT, *vs*. presumptive treatment. The study took place in two different malaria transmission settings in rural Uganda to provide evidence to optimise use of ACTs and mRDTs within programmes of integrated community case management (iCCM) that are currently being scaled up in Uganda and other countries.

## Materials and methods

### Study area

The study was conducted in Rukungiri District, south‐western Uganda, inhabited by the Bahororo and Bakiga ethnic groups, whose main occupation is subsistence farming. Anaemia among under‐fives in south‐western Uganda is estimated at 40%, and in other regions of the country, it ranges from 57% to 74% 11. The district was selected for the study due to its wide altitudinal range between 980 and 2160 m above sea level, resulting in diverse malaria transmission patterns within the same district. Two subcounties with contrasting malaria transmission were selected as trial sites: Bwambara subcounty, a lower altitudinal area bordered by Lake Edward, which is meso‐endemic for malaria with moderate‐to‐high transmission, and Nyakishenyi subcounty, an epidemic‐prone highland area with low transmission, which is hypo‐endemic for malaria.

All villages in the two subcounties were invited to participate in the trial: 63 villages in Bwambara, ranging between 981–1203 m, and 64 villages in Nyakishenyi, ranging between 1064–2157 m. A public meeting was held in each eligible community to explain the purpose of the research, and community members were asked to identify 2–3 CHWs per village for training. Written informed consent to take part in the trial was sought from community leaders and all CHWs selected for training, of whom more than 50% had previously worked in the home‐based management of fever (HBMF) programme that was defunct at the time of the study.

### Trial design

A cluster‐randomized two‐arm trial design was used to compare the effectiveness of mRDTs used by CHWs to diagnose and treat malaria with ACT [intervention] to presumptive diagnosis and treatment of malaria with ACT [control]. The unit of randomisation was the village, with all CHWs in the same village receiving the same type of training. Within each transmission area (subcounty), villages were randomly allocated, using a random number table in Epi Info, to either the intervention (mRDT) or control (presumptive treatment) arm.

**Figure 1:**
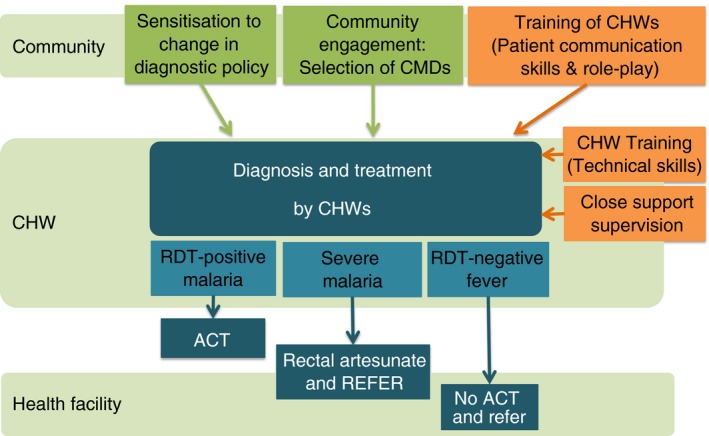
Design of an intervention to support use of malaria diagnostic tests (mRDTs) by community health workers in Uganda.

Community health workers in both arms of the trial were trained in recognising signs and symptoms of malaria, how to administer antimalarial treatment and when to refer (Figure 1, Table 1). Half of the villages were randomized to receive additional training in malaria diagnosis using mRDTs (intervention villages) and trained to only give antimalarial treatment with ACT after a positive test result, with the aim to increase the proportion of children receiving appropriately targeted treatment.

**Table 1:** Training and supporting interventions for community health workers in the intervention and control arms

	Control arm	Intervention arm
3‐ to 4‐day training workshop		
Reasons for policy change to mRDT testing before malaria treatment		✓
How to perform and interpret a mRDT		✓
What to do for patients with a negative mRDT result		✓
How to treat a child with malaria	✓	✓
How to recognise and treat a child with signs of severe illness, and when to refer	✓	✓
How to recognise a child with fevers caused by other illnesses, and when to refer	✓	✓
Role‐play and communication skills	✓	✓
Supporting interventions for trained CHWs		
Training certificate with Ministry of Health logo	✓	✓
ACTs: age‐appropriate pre‐packed blister packs with pictorial labelling	✓	✓
Pictorial job aids	✓	✓
Close support supervision after training – for first 6 months only	✓	✓
Bicycle, T‐shirt, monthly kerosene allowance and soap	✓	✓
Supporting interventions in the community		
Community‐directed selection of CHWs by popular vote	✓	✓
Community sensitisation about mRDTs (community meetings, churches, women's groups)	✓	✓

### Intervention

Participatory training workshops in malaria case management were held for CHWs in each arm, based on a trainer's manual and accompanying set of pictorial job aids (available from www.actconsortium.org/RDThomemanagement). Training workshops lasted 3 days for CHWs in the control arm and 4 days for CHWs in the mRDT intervention arm. The latter received an additional 1‐day training to cover the rationale for diagnostic testing in febrile patients, performing an mRDT and interpretation of the test result based on training materials from WHO 12. CHWs in the intervention arm were trained to treat children with a mRDT‐positive test and symptoms of uncomplicated malaria with artemether–lumefantrine; and to treat mRDT‐positive children with danger signs with rectal artesunate as a pre‐referral treatment, and refer these children to the nearest health unit. CHWs were also trained to refer mRDT‐negative children if the child either had danger signs or other specific symptoms where examination and treatment at a health facility would be beneficial.

Community health workers in the control villages were trained to either dispense artemether–lumefantrine or rectal artesunate based on presumptive diagnosis of malaria, according to signs and symptoms. The training, support materials and procedures were identical in every respect except for the method of diagnosis to be used. In both arms, CHWs were trained in signs for referral (Box 1), completion of treatment records, stock management and preparation of blood slides for reference microscopy. Training encouraged interactive discussion and reflective practice, supported by role‐play to practice communication skills necessary to obtain clinical history and explain outcome of diagnosis, treatment given and referral advice.

Box 1 **Referral guidelines for community health workers**

**Table:** 

(a) Danger signs for urgent referral	(b) Other signs for referral
*Refer using emergency referral form if child shows any of the following symptoms:* Convulsions or fits now or within the past 2 days Coma/loss of consciousness Patient is confused or very sleepy; cannot be woken Extreme weakness – unable to stand or sit without support Very Hot – with temperature of 38.5°C or more Very Cold – with temperature of 35.0°C or less Vomiting everything – cannot keep down food or drink Not able to drink or breastfeed Severe anaemia – very pale palms, fingernails, eyelids Yellow eyes Difficulty in breathing Severe dehydration – sunken eyes, sunken fontanelle, skin pinch, coated tongue	*Refer using ordinary referral form if child shows any of the following symptoms:* Fever that has lasted for more than 7 days Fever with measured temperature of 37°C or more and a negative malaria test result Vomiting and diarrhoea Blood in faeces or blood in urine Pain when passing urine, or frequent urination Wound or burns Skin abscess Painful swelling or lumps in the skin Ear infection (runny ear or child pulling at ear) Sticky or red eyes Fever in a baby <4 months old
*If RDT result is positive*	*If RDT result is positive*
Treat child (if older than 2 months) with rectal artesunate suppository prior to referral.	Treat child (if older than 4 months) with artemether–lumefantine tablets prior to referral.

### Supporting interventions

Recognising that CHWs may need to broker the change in diagnostic practice with communities, the training included practice in communication skills necessary to explain the rationale for diagnostic testing, result of the mRDT test and treatments given. In addition, community sensitisation on diagnostic testing for malaria was carried out throughout the study area prior to the trial (including communities in both arms). The key messages were that not all fevers are malaria and hence a diagnostic test was advisable before treatment with ACT; and a quick test (mRDT) could test for malaria, and these tests were available from government health facilities and trained CHWs in the intervention villages.

For the first 6 months of implementation (July–December 2010), CHWs in both arms received additional close support supervision by project staff through parish‐level CHW meetings to assist adoption of the new procedures into everyday practice, to promote accurate and complete record keeping and to give advice on how to handle difficult situations that could affect adherence, for example caretakers insisting on treatment even after a negative RDT result. At the end of the 6 months, supervision was scaled back to periodic contacts when CHWs came to collect their supplies and a monthly allowance for paraffin and soap intended to support good hygiene and work in the evening/night (15 000 Uganda Shilling, approximately 4.50 USD). CHWs were also provided with pictorial job aids, training certificates, bicycles and T‐shirts at the start of the programme. Although CHWs in Uganda are volunteers, expected to work for free, programmes often provide small ‘refunds’ or tokens, which are generally expected as compensation.

### Evaluation of outcomes

Data on diagnosis and treatment were recorded prospectively on all children seeking treatment for fever in carbon‐copy duplicated CHWs' registers, provided for this purpose. The treatment register for CHWs in the control arm was similar to the intervention arm except for data on mRDT result. CHWs in both arms were trained on how to prepare a thick blood film for every child with fever who consulted them, for later microscopy, subject to verbal assent given by the caretaker accompanying the child. Treatment decisions made by CHWs were validated by light microscopy on the reference blood slide collected at the time of consultation, to compare appropriate targeting of ACTs between arms over a 12‐month period (January–December 2011). The primary endpoint was the proportion of febrile children with malaria receiving appropriately targeted treatment with a first‐line antimalarial, a composite outcome defined as: children with microscopically‐confirmed presence of parasites in a peripheral blood smear (slide positive) who received either artemether–lumefantrine or rectal artesunate, and febrile children with no malaria parasites (slide negative) who did not receive either of these two artemisin‐based treatments (denominator: all consultations for fever or history of fever). The other co‐primary outcome was prompt effective treatment defined as the proportion of febrile children who received appropriately targeted ACT treatment from a CHW within 24 h of onset of malaria symptoms, while the secondary outcome was over‐prescription defined as the proportion of children not parasite‐positive (slide negative) who received inappropriate ACT treatment from CHW (denominator: all children with a negative research slide).

### Sample size estimation

Based on an observed prevalence of malaria of 50% and inter‐cluster variation *k* = 0.35 in the moderate‐to‐high transmission area during the period of close support supervision, a minimum sample size of 6 patients per cluster (180 patients per arm) was required to demonstrate a 50% increase in appropriately targeted treatment with use of mRDTs from 50% to 75%, with 90% power at the 5% significance level, in a cluster‐randomised trial with 30 clusters per arm. The equivalent sample size in the low transmission area, with 30% malaria prevalence, 32 clusters per arm and inter‐cluster variation *k* = 0.45, to demonstrate an increase from 30% to 65% was 2 patients per cluster (128 patients per arm). Taking into account the lower proportion of cases seeking treatment within 24 h of onset of symptoms (89% and 83% in each site), sample size requirements to detect a comparable increase in prompt appropriately targeted treatment were 9 patients and 3 patients per cluster (270 and 192 per arm), in the high‐to‐moderate and low transmission areas respectively.

### Laboratory methods

A finger‐prick blood sample was collected by the CHW at the time of consultation, a thick blood film for microscopy prepared, and mRDT (First Response^®^ Ag. P. falciparum (HRP2) Card test, www.PremierMedCorp.com; intervention arm only) performed. The mRDTs passed independent quality‐assurance batch testing by WHO‐FIND, Institut Pasteur, Cambodia. Thick blood smears were haemolysed and stained with 10% Giemsa for 10 min and examined under light microscopy with oil immersion objective lens (×100). Asexual parasites were counted against 200 white blood cells and parasite densities calculated assuming a standard white blood cell count of 8000/*μ*l blood. A blood smear was declared negative if no parasites were detected in 100 high power fields. Reference slides included in the analysis of the primary endpoint were subjected to two independent readings by light microscopy. The second reading was performed by experienced technicians at Vector Control Division, Ministry of Health in Kampala, blinded to initial results. Any discrepant results between the first and second readings were resolved by a third technician blinded to all previous results. As the number of consultations in Bwambara (moderate‐to‐high transmission area) exceeded the sample size requirements, a subsample of blood slides was randomly selected for analysis of the primary endpoint. To reflect seasonal variation in the prevalence of malaria, two slides were randomly sampled per month from each village during the main evaluation period from January to December 2011 for double reading (a total of 24 slides per cluster). Malaria was less common in Nyakashenyi, with fewer consultations, and thus, all available slides were re‐examined by expert microscopy.

### Statistical methods

**Figure 2:**
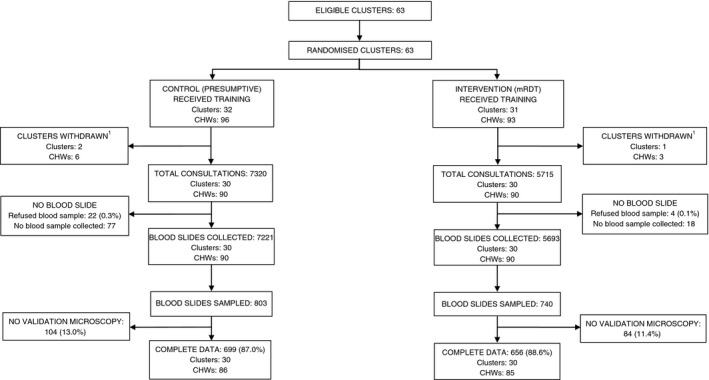
Trial profile, moderate‐to‐high transmission area. ^1^After training, CHWs in villages located close to the border of the study area started to receive febrile children from outside the study district; these villages were subsequently withdrawn from the trial.

Data were analysed as intention‐to‐treat, with analysis conducted separately for each transmission setting. Due to the withdrawal of some clusters from the study after randomisation in the moderate‐to‐high transmission setting (Figure 2), data were analysed as modified‐intention‐to‐treat. To account for cluster‐randomised design, a random effects logistic regression model was used to estimate intervention effects and 95% confidence intervals; a quadrature check performed to confirm model was a reliable fit to the data, and likelihood ratio test used to assess statistical significance 13. The analytical plan was approved by an independent data safety and monitoring board, prior to analysis. Data were double‐entered and verified using Microsoft Access 2007 (Microsoft Inc., Redmond, USA) and analysed using STATA version 12 (STATA Corporation, College Station, Texas, USA).

### Ethical approval

This study was approved by the Uganda National Council for Science and Technology and the London School of Hygiene & Tropical Medicine Ethics Committee. Patients refusing an mRDT test received presumptive treatment. The study was registered with ClinicalTrials.gov. Identifier NCT01048801 on 13 January 2010.

## Results

**Figure 3:**
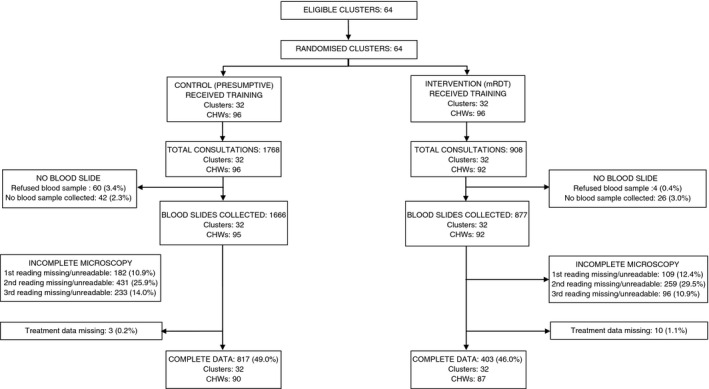
Trial profile, low transmission area.

To account for seasonal variations in malaria risk, data were analysed for a 12‐month period of routine implementation (January–December 2011), after the training and 6‐month period of close support supervision had ended. During 2011, a total of 13 035 children were seen by CHWs in 63 villages in Bwambara subcounty (the moderate‐to‐high malaria transmission area), with 5715 children seen by CHWs in the mRDT intervention arm and 7320 in the control arm (Figure 2). In Nyakashenyi subcounty (the low transmission area), a total of 2676 children were registered by CHWs in 64 villages during the same period: 908 and 1768 in mRDT and presumptive arm, respectively (Figure 3). Most patients were willing to provide a blood sample, in all study arms, with few refusals.

**Figure 4:**
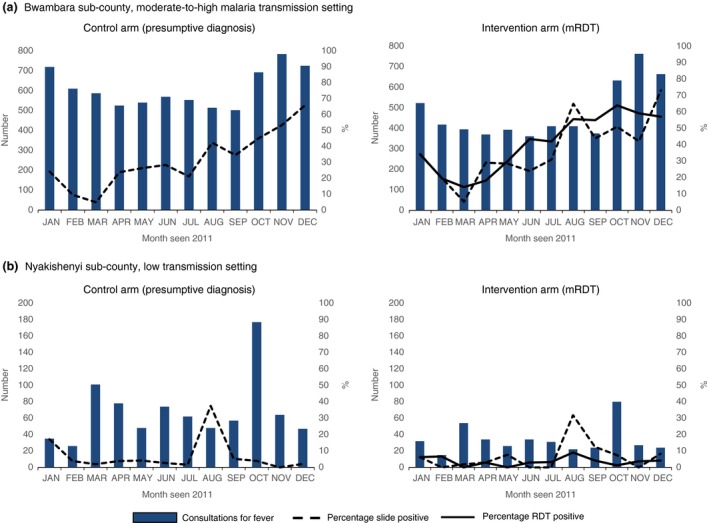
Consultations for fever, malaria blood slide and rapid diagnostic test positivity by month in two areas of contrasting malaria transmission intensity in south‐western Uganda: January–December 2011. (a) Bwambara subcounty, moderate‐to‐high transmission setting. (b) Nyakishenyi subcounty, low transmission setting.

In both transmission settings, the number of consultations in the control arm usually exceeded those in the mRDT arm, although the seasonal pattern was similar across arms (Figure 4). In Bwambara subcounty, changes in mRDT positivity mirrored changes in blood slide positivity. In Nyakashenyi subcounty, slide positivity was generally <10%, but increased to 35.7% in August 2011; mRDT positivity reached a peak of 9% in the same month.

### Effect of intervention on appropriately targeted treatment: moderate‐to‐high transmission setting

Of the 12 914 blood slides collected by CHWs in Bwambara subcounty, 1543 were sampled for validation of the treatment decision by expert microscopy (Figure 2). Appropriately targeted treatment was evaluated in patients for whom data on two or more independent slide readings were available: 699 patients in the control arm and 656 patients in the mRDT intervention arm, comprising 87.0% and 88.6% of the slides sampled in each arm, respectively. CHW and patient characteristics were generally similar between the two arms (Table 2). While over 80% of patients had sought treatment within 24 h of onset of symptoms, there was greater delay in the mRDT intervention villages than controls. Slide positivity was also higher in the mRDT intervention arm, 37.8% *vs*. 30.2% control (Table 3), with 37.6% of children in the intervention arm testing mRDT positive. The sensitivity and specificity of mRDT result against expert microscopy were 72.1% and 83.3%, respectively, with a positive predictive value (PPV) of 72.4% and negative predictive value (NPV) of 83.1% in this area of moderate‐to‐high transmission (Table 5).

**Table 2:** Characteristics of clusters and community health workers in the intervention and control arms

		Moderate‐to‐high transmission		Low transmission	
		Control arm	Intervention arm	Control arm	Intervention arm
		Frequency (%)	Frequency (%)	Frequency (%)	Frequency (%)
Number of participating villages		31	32	32	32
Total number of CHWs		90	90	96	96
CHWs characteristics^a^					
Total CHWs interviewed		74	81	21	17
Age in years	Median	39	40	41	40
Sex	Male	24 (32.4)	26 (32.1)	6 (28.6)	2 (11.8)
	Female	50 (67.6)	55 (67.9)	15 (71.4)	15 (88.2)
Highest level of school attendance	Never	1 (1.6)	6 (6.4)	0 (0)	0 (0)
	Primary	40 (64.5)	46 (59.0)	11 (52.4)	11 (64.7)
	Secondary	20 (33.9)	21 (33.3)	10 (47.6)	6 (35.3)
	Tertiary	0 (0)	1 (1.3)	0 (0)	0 (0)
CHW before	Yes	47 (65.3)	39 (52.7)	14 (66.7)	7 (41.7)
	No	25 (34.7)	35 (47.3)	7 (33.3)	10 (58.8)
Involvement in health activities before	Yes	21 (30.0)	31 (45.6)	5 (26.3)	5 (31.3)
	No	49 (68.1)	37 (50.0)	14 (66.7)	11 (64.7)
Elevation (metres above sea level)	Mean (SD)	1131.21 (184.9)	1086.0 (53.7)	1829.8 (110.0)	1839.0 (120.9)
Patient characteristics^b^					
Total number of children seen by CHW		699	656	817	403
Age in years	Mean (SD)	2.8 (1.5)	2.6 (1.4)	2.6 (1.4)	2.4 (1.5)
Sex	Male	359 (51.7	335 (51.1)	434 (53.2)	198 (49.3)
	Female	336 (48.3)	320 (48.9)	382 (46.8)	204 (50.7)
Slept under a mosquito net the previous night	Yes	639 (92.7)	567 (87.4)	737 (91.1)	351 (87.8)
	No	50 (7.3)	82 (12.6)	72 (8.9)	49 (12.3)
Resident in same village as CHW	Yes	644 (92.5)	599 (91.6)	732 (89.8)	331 (82.5)
	No	52 (7.5)	55 (8.4)	83 (10.2)	70 (17.5)
Time of presentation to CHW after of onset of symptoms	Within 24 h	641 (93.4)	544 (84.6)	698 (88.8)	319 (80.2)
	More than 24 h	45 (6.6)	99 (15.4)	88 (11.2)	79 (19.8)

a Data missing in moderate‐to‐high transmission setting, for highest level of school attendance: 21 (13 control, 8 intervention); previous CHW status: 9 (2 control arm, 7 intervention); involvement in health activities before: 17 (4 control, 13 intervention). Data missing in low transmission setting, for involvement in health activities before: 3 (2 control, 1 intervention).

b Data missing in moderate‐to‐high transmission setting, for age: 4 (2 control, 2 intervention); sex: 5 (4 control, 1 intervention); net use: 17 (10 control, 7 intervention); resident in same village 5 (3 control, 2 intervention); onset of symptoms: 26 (13 control, 13 intervention). Data missing in low transmission setting, for age: 14 (5 control, 9 intervention); sex: 2 (1 control, 1 intervention); net use: 11 (8 control, 3 intervention); resident in same village:4 (2 control, 2 intervention); onset of symptoms: 36 (31 control, 5 intervention).

**Table 3:** Diagnosis and treatment of malaria by CHWs in Bwambara subcounty, January–December 2011: moderate‐to‐high transmission setting

Diagnosis and treatment	Control arm 31 clusters	Intervention arm 32 clusters		
	Frequency (%)	Frequency (%)		
Patients with complete data for primary endpoint	699	656		
Blood slide positive	211 (30.2)	248 (37.8)		
mRDT positive^a^	n/a	243 (37.6)		
Malaria treatment prescribed				
Artemether‐lumefantrine tablets (AL)	692 (99.0)	244 (37.2)		
Rectal artesunate suppository	3 (0.4)	2 (0.3)		
Neither AL nor rectal artesunate	4 (0.6)	410 (62.5)		
Malaria treatment by infection status				
Blood slide negative, received no ACT^b^	4 (0.8)	341 (83.4)		
Blood slide negative, received ACT	484 (99.2)	67 (16.4)		
Blood slide positive, received ACT	211 (100.0)	179 (72.2)		
Blood slide positive, received no ACT	0 (0.0)	69 (27.8)		
**Trial endpoints**	***n*** **(%)**	***n*** **(%)**	**Odds ratio (95% CI)**	***P*** **‐value**
Over‐prescription				
Proportion of blood slide negative patients receiving ACT^b^	484 (99.2)	67 (16.4)	0.0013 (0.0004–0.0039)	<0.001
Appropriately targeted treatment				
Proportion of febrile patients receiving appropriately targeted malaria treatment with ACT	215 (30.8)	520 (79.3)	9.71 (6.83–13.80)	<0.001
Prompt and appropriately targeted treatment				
Proportion of febrile patients receiving appropriately targeted treatment within 24 hours of onset of symptoms^c^	195 (28.1)	433 (67.0)	5.92 (4.15–8.45)	<0.001

a Ten patients missing RDT results.

b ACT defined as receiving either artemether‐lumefantrine or rectal artesunate.

c Fifteen missing data on time of treatment (five control, 10 intervention).

**Figure 5:**
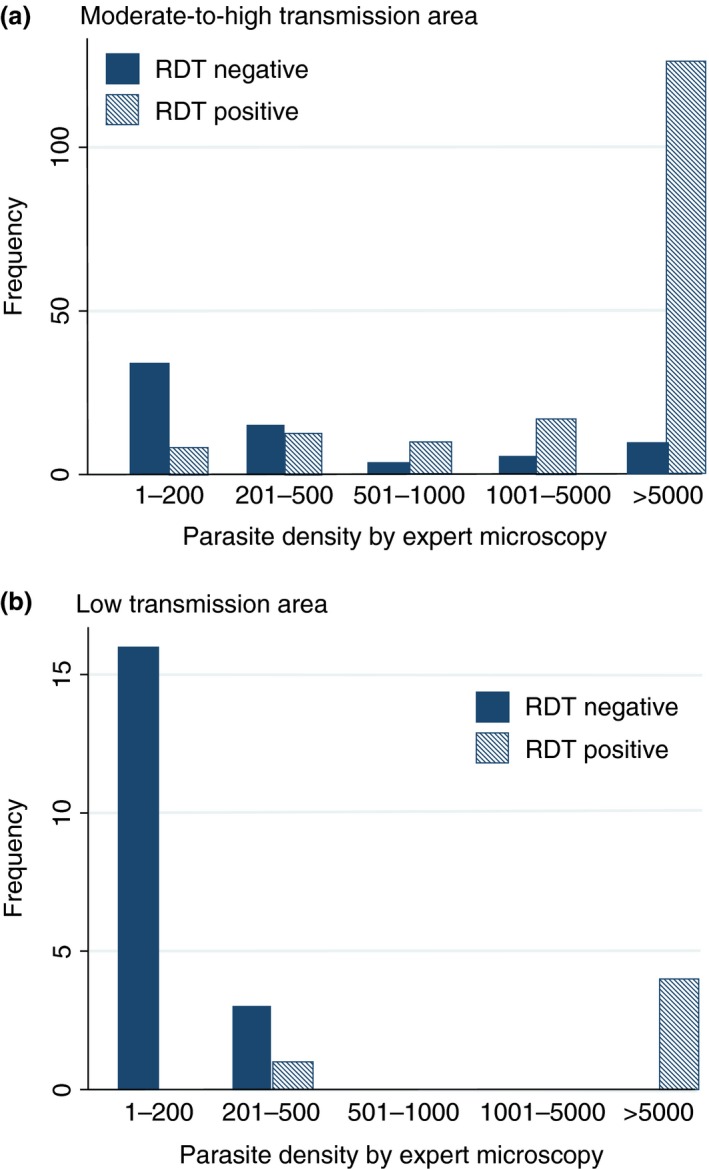
Rapid diagnostic test results performed and read by community health workers in relation to parasite density measured by expert light microscopy (Data are shown for children with parasites detected in a thick blood film). (a) Moderate‐to‐high transmission area. (b) Low transmission area.

Among febrile children seen by CHWs in the mRDT intervention arm, 37.5% were treated with either artemether–lumefantrine or rectal artesunate (hereafter referred to collectively as ACT) compared with almost 100% of children seen by CHWs using presumptive diagnosis (Table 3). Only 16.4% of slide‐negative children in the mRDT intervention arm received an ACT, a significant reduction in over‐prescription compared with the control arm, *P* < 0.001. Sixty‐nine of the patients in the mRDT intervention arm who did not receive ACT treatment were slide positive, of whom 35 (51%) had parasite densities ≤200 parasites per *μ*l (Figure 5a). Use of mRDTs was thus associated with a significant overall improvement in the targeting of ACT treatments, such that in this area of moderate‐to‐high transmission, 79.3% of children in the mRDT arm received appropriately targeted treatment compared with 30.8% in the control arm (odds ratio 9.71, 95% CI 6.83–13.80; *P* < 0.001). Despite greater delay in seeking treatment in the mRDT arm, the proportion of children who received appropriately targeted treatment within 24 h was also significantly higher in the intervention arm (67.0% *vs*. 28.1%; odds ratio 5.92, 95% CI 4.15–8.45; *P* < 0.001).

### Effect of intervention on appropriately targeted treatment: low transmission setting

A total of 2543 blood slides were collected by CHWs in Nyakashenyi subcounty, of which two or more independent slide readings were available for 49.0% and 47.1% of slides in the control and mRDT arm, respectively (Figure 3). Patient records missing treatment data were few. Appropriately targeted treatment was thus evaluated in the 817 and 403 patients with complete data in the control and mRDT intervention arms, comprising 46.2% and 44.4% of consultations in each arm, respectively. Reasons for data loss included poor quality of the slides prepared by CHWs and missing data on validation microscopy. CHW and patient characteristics were similar between the two arms, although, as in the high transmission area, there was slightly greater treatment delay in mRDT intervention villages than controls; 80.2% *vs*. 88.8%, respectively, had sought treatment within 24 h of onset of symptoms (Table 2). Slide positivity was 5.7% overall, and similar in the two arms (Table 4), with 3.0% of children in the intervention arm reported to be mRDT positive. The sensitivity of mRDT result against microscopy was low (20.8%), and specificity was 98.1% (Table 5). PPV in this low transmission setting was 41.7%, and NPV, 95.1%.

**Table 4:** Diagnosis and treatment of malaria by CHWs in Nyakishenyi subcounty, January–December 2011: low transmission setting

Diagnosis and treatment	Control arm 32 clusters	Intervention arm 32 clusters		
	Frequency (%)	Frequency (%)		
Patients with complete data for primary endpoint	817	403		
Blood slide positive	46 (5.6)	24 (6.0)		
mRDT Positive^a^	n/a	12 (3.0)		
Malaria treatment prescribed				
Artemether‐lumefantrine tablets (AL)	773 (94.6)	22 (5.5)		
Rectal artesunate suppository	18 (2.2)	6 (1.5)		
Neither AL nor rectal artesunate	26 (3.2)	375 (93.1)		
Malaria treatment by infection status				
Blood slide negative, received no ACT^b^	22 (2.9)	357 (94.2)		
Blood slide negative, received ACT	749 (97.2)	22 (5.8)		
Blood slide positive, received ACT	42 (91.3)	6 (25.0)		
Blood slide positive, received no ACT	4 (8.7)	18 (75.0)		
**Trial endpoints**	***n*** **(%)**	***n*** **(%)**	**Odds ratio (95% CI)**	***P*** **‐value**
Over‐prescription				
Proportion of blood slide negative patients receiving ACT	749 (97.2)	22 (5.8)	0.00022 (0.00004–0.00125)	0.002
Appropriately targeted treatment				
Proportion of febrile patients receiving appropriately targeted malaria treatment with ACT	64 (7.8)	363 (90.1)	162.9 (83.0 – 319.6)	<0.001
Prompt and appropriately targeted treatment				
Proportion of febrile patients receiving appropriately targeted treatment within 24 hours of onset of symptoms^c^	49 (6.0)	287 (72.1)	40.3 (28.1 ‐ 57.9)	<0.001

a One patient missing mRDT result.

b ACT defined as receiving either artemether‐lumefantrine or rectal artesunate.

c Nine missing data on time of treatment (four control, five intervention).

**Table 5:** Sensitivity and specificity of malaria rapid diagnostic tests (mRDTs) performed by community health workers in two areas of contrasting transmission intensity

Result reported by CHW^a^	Moderate‐to‐high transmission setting			Low transmission setting		
	Expert microscopy^b^		Predictive value of mRDT, %	Expert microscopy^b^		Predictive value of mRDT, %
	Positive (%)	Negative (%)		Positive (%)	Negative (%)	
mRDT positive	176 (72.1)	67 (16.7)	72.4	5 (20.8)	7 (1.9)	41.7
mRDT negative	68 (27.9)	335 (88.3)	83.1	19 (79.2)	371 (98.1)	95.1
Total samples examined	244	402		24	378	

a Missing data on RDT result: 10 in high transmission setting; 1 in low transmission setting.

b Result of blood slides double‐read by two independent microscopists, with discrepant findings resolved by a third independent reader; all blind to RDT result.

When compared with malaria status by microscopy, 5.8% of slide‐negative children in the mRDT intervention arm received an ACT, a significant reduction in overprescription compared with control arm, *P* = 0.002 (Table 4). However, of the 24 patients who were slide positive, only 6 had received ACT treatment; 17 of the 18 that were untreated had parasite densities ≤200 parasites per *μ*l (Figure 5b). Overall, use of mRDTs was associated with a dramatic improvement in the targeting of ACT treatments; 94.2% of children in the mRDT intervention arm received appropriately targeted treatment compared with 25.0% on the control arm (odds ratio 162.9, 95% CI 83.0–319.6; *P* < 0.001). The proportion of children who received appropriately targeted treatment within 24 h was also significantly higher in the intervention arm (72.1% *vs*. 6.0%, *P* < 0.001).

## Discussion

This study confirms that use of mRDTs by CHWs can reduce the number of ACTs used, as documented in previous studies 10, 14, 15, and additionally demonstrates that mRDTs can substantially improve the targeting of ACT treatment to children with malaria in community‐based treatment programmes. With use of mRDT‐based diagnosis, the proportion of children receiving appropriately targeted ACT treatment from CHWs (consistent with their true malaria infection status as determined by later expert microscopy) exceeded 80% in both transmission settings; a significant increase over presumptive treatment (*P* < 0.001 in both sites). The effect was more marked in the low transmission area where more than 90% of the patients received appropriately targeted treatment in the mRDT arm. This could be attributable to the combination of high specificity of mRDT in a setting of low and unstable malaria transmission, where nearly 100% of mRDT negatives were negative by light microscopy, and high adherence to mRDT results by CHWs. In contrast, where CHWs followed presumptive diagnosis, more than 90% of patients subsequently found to be slide negative had received an unnecessary ACT treatment. This concurs with previous reports that overuse of ACTs is likely to be higher in areas with low malaria prevalence 16, 17. A high degree of adherence to test results by CHWs has frequently been observed in studies among CHWs, in contrast to the less favourable reports from research among health facility‐based providers and some other community programmes 18, 19, 20, 21, 22, 23. Factors which may have contributed to high adherence in our trial were the comprehensive and participatory training that CHWs received, including clear guidance on referral criteria and how to handle RDT‐negative patients, and community meetings to create awareness that not all fevers are due to malaria and explain the rationale for diagnostic testing at community level. Furthermore, the use of job aids, previously found to be an important aid for CHWs to interpret and adhere to mRDT results 23, and supportive supervision during the immediate post‐training period, may have sustained the awareness and adherence to guidelines 24 and enhanced CHWs self‐esteem and confidence in what they were doing. Educative interaction between caretakers and health workers has been shown to be a major factor for community acceptability of RDT use 25. Close proximity of CHWs to other members of the community, coupled with community sensitisation and the communication training provided to CHWs as part of the intervention, may also have facilitated high adherence to mRDT results by CHWs.

More than 80% of patients with fever in the intervention and control arms in both transmission settings consulted CHWs for treatment within 24 h after onset of symptoms, which is much higher than has previously been observed at health facilities in Uganda 11 and elsewhere 26. These data show that CHWs provide an opportunity for improving the speed of treatment seeking for febrile illnesses in rural areas, where poor access to health facilities, lack of drugs, perceived high cost and unfriendly attitude of health workers often result in delayed treatment 27, 28, 29, 30, 31, 32 and illustrate the role of community‐based treatment programmes in increasing access to prompt effective treatment of malaria.

The sensitivity of mRDTs in this study was lower than has been previously observed among CHWs 23, particularly in the low malaria area, which could be due to the large proportion of low parasite density infections observed in those patients who were untreated, and which may therefore have been undetected by mRDT 33, 34. Blood slides were prepared by CHWs, which was found to be a particular limitation in our study and others 14, and reduced the number of samples examined by microscopy and thus available for analysis, especially in the low transmission area. Nonetheless, the characteristics of patients included in the final analysis and CHWs were comparable between arms and we do not consider our findings could be attributed to selection bias. The mRDTs used passed independent quality‐assurance batch testing, and we can confidently exclude test performance as a reason for low sensitivity. As the majority of false negatives were low‐density infections, we can also exclude the possibility that this was due to a prozone effect; though the frequency of HRP‐2 deletions in this population is unknown 35. Other possible causes for false‐negative mRDT results include operator error, especially where a CHW works under poor lighting conditions and/or at night 35. Nonetheless, as missed treatment was frequently observed in low‐density infection, below the acknowledged limit of detection of most mRDTs, we consider this to be the most likely cause for this observation. False positives were higher in the high transmission area than in the low transmission area, as would be expected due to persistent antigenaemia in individuals recently infected by malaria in hyperendemic areas 36.

The benefits of using mRDTs to improve targeting of ACTs and limit their overuse need to be weighed against the risks of not treating an infected child 37. Among semi‐immune children with low parasite counts in the moderate‐to‐high transmission area, the probability that the fever is attributable to malaria is low 38, 39, and antimalarial treatment is not necessarily required. However, missed treatment in non‐immune subjects in the low transmission area is of greater potential concern. A limitation of this study was that we did not follow up patients to see if they recovered so we do not know the clinical outcome in those who missed receiving treatment when they were mRDT negative but proved to be parasite‐positive on microscopy. Poor sensitivity of HRP‐2‐based mRDTs has previously been reported from other areas of low transmission 33, 34, and more sensitive point‐of‐care diagnostic techniques, of low cost and suitable for routine use in these low transmission settings, are needed. Thus, before scaling up mRDT use by CHWs in low transmission areas, we would suggest that safety studies with follow‐up data on patients who missed treatment due low sensitivity of current mRDTs be conducted to adequately assess the risk of non‐treatment of low‐density infections in non‐immunes. It is conceivable that missed treatments are more frequent in community‐based programmes than in other health services using mRDTs, as patients may be more likely to seek care at an earlier stage in their illness. Parasite density among patients presenting to CHWs may therefore be lower than in patients who first present to health facilities, and a greater proportion of them may have an infection below the detection threshold of current mRDTs, irrespective of local transmission intensity. Encouraging patients with no symptoms other than fever, who test mRDT negative, to return to the CHW for re‐evaluation and retesting whether symptoms do not improve, could help mitigate the risk of not treating an infected child.

## Conclusion

The findings presented here underscore the value of mRDT‐based diagnosis of malaria in reducing overuse of ACTs in community case management and should be of particular interest to malaria programme managers and policymakers, nationally, regionally and internationally as countries scale up iCCM 40. Equipping CHWs with mRDTs markedly improved the appropriate targeting of ACT treatment. With increased financing for malaria and large‐scale control, the prevalence of malaria should reduce and the potential role of mRDTs in targeting malaria treatment and disease surveillance will become ever more critical 41 at all levels of health service delivery, including community level. Use of mRDTs and diagnostic algorithms has the additional potential to also improve detection and treatment of non‐malaria fevers. Our data show mRDT use was beneficial in improving the targeting of ACT use in both moderate‐to‐high and low transmission areas and that a widespread adoption of mRDTs at community level can reduce misdiagnosis and inappropriate treatment of febrile illness in all malaria transmission settings. Nonetheless, mRDTs have several limitations that may reduce their utility in low transmission settings because they do not reliably detect low‐density parasitaemia. Studies have shown that in infants, parasitaemia is synonymous to clinical malaria 42. Therefore, missed treatments due to the low sensitivity of current mRDTs to low‐density infections (as reported here and in other areas of low transmission 34) are a concern and suggest that despite the potential cost savings from reduced use of ACTs, presumptive diagnosis and treatment of all fevers as malaria may remain advisable in malarious areas where populations have little or no immunity, until such time as more sensitive diagnostic assays become widely available.
